# Organ perfusion during partial REBOA in haemorrhagic shock: dynamic 4D-CT analyses in swine

**DOI:** 10.1038/s41598-022-23524-y

**Published:** 2022-11-05

**Authors:** Yosuke Matsumura, Akiko Higashi, Yoshimitsu Izawa, Shuji Hishikawa

**Affiliations:** 1 Department of Intensive Care, Chiba Emergency Medical Center, 3-32-1 Isobe, Mihama, Chiba City, 261-0012 Japan; 2 Department of Emergency and Critical Care Medicine, Chiba University Graduate School of Medicine, Chiba City, Chiba Japan; 3 Department of Emergency and Critical Care Medicine, Jichi Medical University, Shimotsuke City, Tochigi Japan; 4 Center for Development of Advanced Medical Technology, Jichi Medical University, Shimotsuke City, Tochigi Japan

**Keywords:** Translational research, Experimental models of disease

## Abstract

Resuscitative endovascular balloon occlusion of the aorta (REBOA) increases proximal blood pressure while inducing distal ischemia of visceral organs. The evaluation of distal ischemia severity during REBOA is a prerequisite for safe resuscitation of haemorrhagic shock patients with REBOA. We evaluated changes in blood flow and organ perfusion due to the degree of occlusion using dynamic 4D-computed tomography (CT). We compared the results with those of a previous study on euvolemic status. Delayed enhancement of the inferior vena cava (IVC) without retrograde flow was observed in the 4D-volume rendering images in the high-degree occlusion. The time-density curve (TDC) of the liver parenchyma (liver perfusion) and superior mesenteric vein (SMV) demonstrated a decreased peak density and a delayed peak in high-degree occlusion. The change rate of the area under the TDC of the liver and SMV decreased linearly as the degree of occlusion increased (PV, Y = −1.071*X + 106.8, r^2^ = 0.972, P = 0.0003; liver, Y = −1.050*X + 101.8, r^2^ = 0.933, P = 0.0017; SMV, Y = −0.985*X + 100.3, r^2^ = 0.952, P = 0.0009). Dynamic 4D-CT revealed less severe IVC congestion during P-REBOA in haemorrhagic shock than in euvolemia. Analyses of TDC of the liver and SMV revealed a linear change in organ perfusion, regardless of intravascular volume.

## Introduction

Resuscitative endovascular balloon occlusion of the aorta (REBOA) is a minimally invasive aortic occlusion technique used to resuscitate refractory haemorrhagic shock. REBOA increases proximal blood pressure while inducing distal ischemia of visceral organs and lower extremities, which causes inflammatory sequelae　that may be life- or limb-threatening^1–4^. Thus, the search for methods to alleviate organ ischemia is a prerequisite for safe resuscitation with REBOA.

Aortic flow regulation with partial balloon inflation, partial REBOA (P-REBOA), is suggested to mitigate distal ischemia and extend survival^2–5^. P-REBOA is widely used instead of complete REBOA. However, a reproducible and quantitative definition of cutoff intensity is needed to validate its association with organ perfusion. Although P-REBOA has been previously defined using computed tomography (CT) images, this method cannot be used in clinical practice^6–9^. We previously reported that balloon volume could be used as a parameter of the degree of occlusion^10^. Additionally, balloon volume was associated with abdominal organ perfusion as a clinically usable index by analyzing the time-density curve (TDC) of dynamic four-dimensional (4D) CT. The TDC was calculated using the elevation of the density from the baseline according to the time to evaluate the blood flow and organ ischaemia^11^. Density was described in Hounsfield units (HU), and time was described in seconds (s). These two previous studies were done in the euvolemic status.

During haemorrhagic shock, aortic diameter changes significantly^12^. Balloon volume required to occlude the aorta changes proportionally with changes in aortic diameter^13^. We observed that the caudal side of the balloon was initially inflated. The balloon's cross-sectional area at the mid-segment (not the widest part) linearly increased with the balloon volume^10^. However, the cross-sectional area is only evaluated with the CT scan, which is often unavailable in the REBOA use situation. Although the balloon volume could be a clinically available surrogate for the degree of P-REBOA, it has not been verified whether the relationship between organ perfusion and balloon volume reported in euvolemia is also observed during haemorrhagic shock.

We hypothesized that 4D-CT could assess TDC in a haemorrhagic shock model to evaluate abdominal organ perfusion. This study aimed to compare hemodynamic changes during P-REBOA in haemorrhagic shock with those in non-haemorrhagic conditions and evaluate the relationship between balloon volume and visceral organ perfusion.

## Materials and methods

### Overview

This study was conducted in an accredited animal research laboratory (Center for Development of Advanced Medical Technology, Jichi Medical University, Tochigi, Japan). Approval was obtained prior to conducting the study from the Animal Experiment Committee of the Center for Experimental Medicine, Jichi Medical University (authorization no. 17045-01). All methods were performed according to the relevant guidelines and regulations. Healthy female non-pregnant domestic pigs (n = 4) were obtained from Sanesu Breeding Co., Ltd. (Chiba, Japan) and were used in this study. We utilized the experimental model from our previous research in the non-haemorrhagic models to minimize technical error and effects of subjective bias, and introduced the haemorrhagic shock model in this study^11^. We evaluated and compared the results with those of a previous study^11^. The animals were quarantined for a minimum of 7 days and fasted for 24 h with access to water before enrolment in the experimental protocol. At the time of experimentation, the animals were 3–4 months of age and weighed 30–40 kg. The experimental protocol included two phases: animal preparation and dynamic 4D-CT scan with P-REBOA, followed by CT data analysis.

### Animal preparation

The swine were premedicated intramuscularly with 0.06 mg/kg medetomidine (Nippon Zenyaku Kogyo Co., Ltd., Fukushima, Japan), 0.3 mg/kg midazolam (Astellas Pharma Inc., Tokyo, Japan), and 0.08 mg/kg atropine (Mitsubishi Tanabe Pharma Corporation, Osaka, Japan) in the animal cage in the early morning. After confirming sedation and endotracheal intubation in the animal operating room, maintenance anaesthesia consisting of 1–3% sevoflurane was applied. 1% propofol was injected intravenously when necessary since inhalation anaesthesia was unavailable due to breath-holding during the 4D-CT scan lasting for two minutes to minimise the motion artifacts (Maruishi Pharmaceutical Co. Ltd., Osaka, Japan). The animals were mechanically ventilated with tidal volumes of 7–10 mL/kg and a respiratory rate of 10–15 breaths/min, which was sufficient to maintain the end-tidal CO_2_ at 40 ± 5 mmHg. F_I_O_2_ was titrated according to oxygenation during the experimental procedure. The oxygenation target was SpO_2_ at 95–99%, but the F_I_O_2_ was increased at 100% before breath-holding during CT scanning. The swine were placed on a warming blanket set at 39 °C to maintain body temperature.

### Surgical procedures and REBOA placement

After induction of general anaesthesia, the right neck was exposed, and an arterial line was catheterized for proximal pressure monitoring and blood sampling into the right carotid artery. A central venous catheter was then inserted in the right jugular vein. Both groins were exposed and a 10-Fr sheath was placed into the right femoral artery to insert a 7-Fr REBOA catheter (Rescue Balloon®; Tokai Medical Products, Aichi, Japan). The side-arm of the 10-Fr sheath was used for distal pressure monitoring. Acetated Ringer’s solution was infused, targeting a stroke volume variation between 10 and 15%, and a bolus injection was added when the blood pressure dropped. The animals were transferred to a CT scanner (SOMATOM® Definition AS+ [128-slice]; Siemens Healthcare GmbH, Erlangen, Germany) under general anesthesia. A REBOA catheter was placed in the thoracic aorta to maintain balloon position in Zone 1. The REBOA catheter was fixed and the balloon was gradually inflated with close distal pressure monitoring. Total REBOA (100% occlusion) was defined as the complete cessation of distal pulse pressure^10^. Percent balloon volume was defined as the percentage of the balloon volume to the maximum balloon volume, which is used as a parameter of the degree of occlusion during P-REBOA.

### Induction of Haemorrhagic Shock

Using a 10-Fr arterial sheath in the right femoral artery, 30 mL/kg of blood (approximately 40% blood loss), was withdrawn for 20 min exponentially to induce class IV shock. The first half of this volume was removed at 2.15 mL/kg/min for 7 min and the remainder was removed at 1.15 mL/kg/min for 13 min^14^.

### Dynamic 4D-CT

Swine were scanned in the supine position every 20% of maximum balloon volume using a previously described scan protocol^11^. Balloon position was confirmed in Zone 1 by a pre-perfusion scan. For the dynamic 4D-CT, a 600 mg iodine/kg bolus of iopamidol (300 mg iodine/mL Iopaque®; FujiPharma, Tokyo, Japan) was administered intravenously for 30 s through the right jugular vein. The dynamic 4D-CT scan was continued for 112 s from the beginning of the contrast injection. The scanning range was 204 mm from the top of the liver. The 4D-volume rendering (VR) images were generated to visualize the change in blood flow in the great vessels (Ziostation2® PLUS Classic S, Ziosoft Inc., Tokyo, Japan). The blood flow patterns of the 4D-VR were visually analyzed according to the degree of occlusion.

### Analysis of the TDC

The dynamic 4D-CT data were evaluated as previously described^11^. We chose the liver parenchyma and superior mesenteric vein (SMV) as the regions of interest. Liver parenchyma was evaluated as a parameter of liver perfusion, and SMV was evaluated as a parameter of mesenteric perfusion. The TDC was calculated using ImageJ^15^. Because baseline density could vary, the TDC was defined by the elevation of the density from baseline and the time from the initiation of the contrast injection. Density was described in Hounsfield units, and time was described in seconds (s). The area under the TDC (AUTDC) was calculated to evaluate the changes in perfusion caused by changes in the degree of occlusion. The change rate of the AUTDC was assessed with 0% occlusion as a reference and analyzed with linear regression using GraphPad Prism 6.07 for Windows (GraphPad Software Inc., La Jolla, CA, USA).

### Ethical approval

This study was conducted in an accredited animal research laboratory (Center for Development of Advanced Medical Technology, Jichi Medical University, Tochigi, Japan). Institutional Animal Experiment Committee approval was obtained before beginning the study (authorization number 17045-01). This study is reported in accordance with ARRIVE guidelines (https://arriveguidelines.org).

## Results

All experimental animals were anaesthetized and underwent safe surgical procedures. Despite temporary hemodynamic instability, no animals died during the phlebotomy. No critical adverse events were observed during the study. The maximum balloon volume (100%) was 5 mL, 6 mL, 5 mL, and 8 mL, respectively.

**Figure 1:**
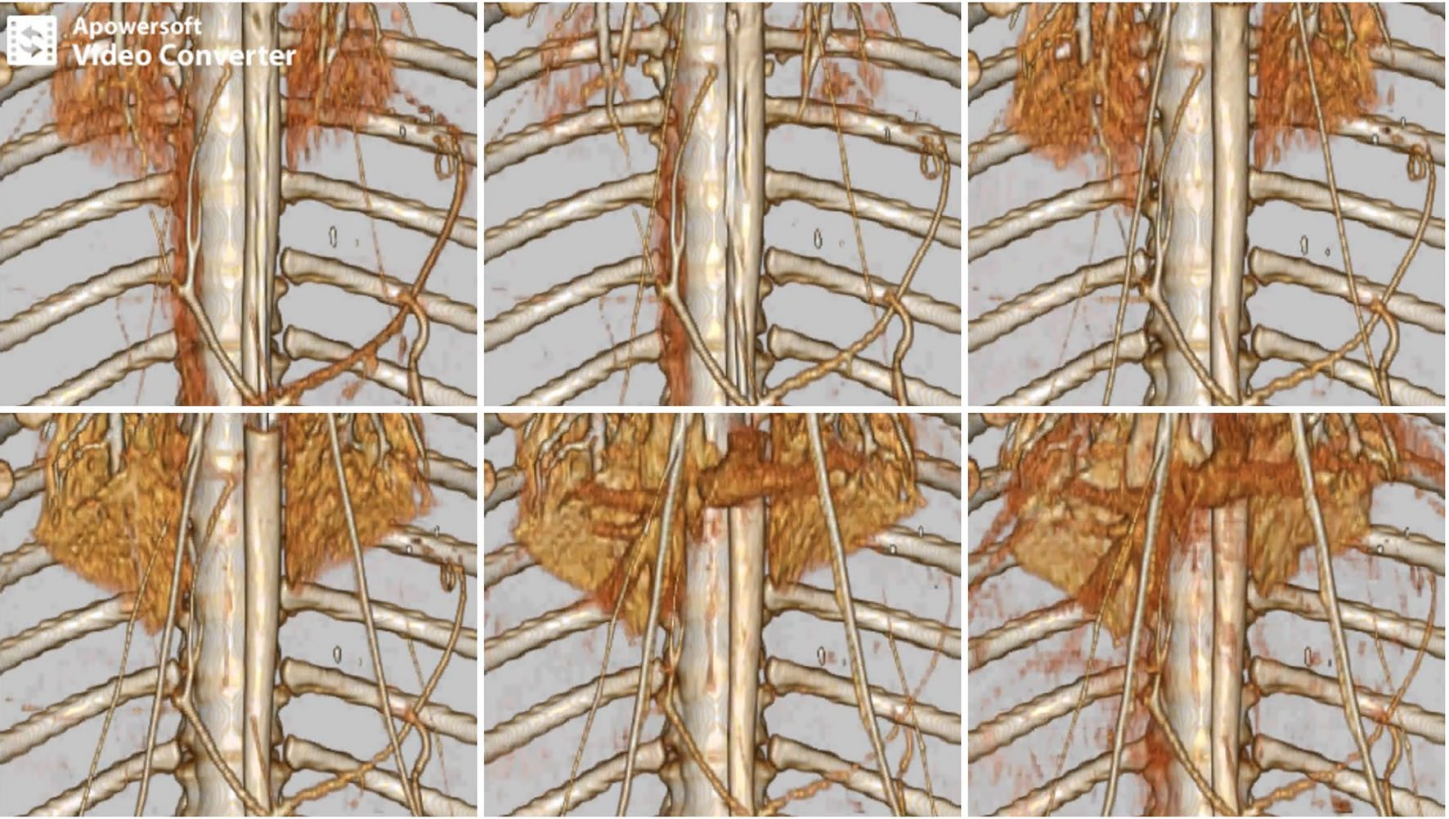
Volume-rendering images of dynamic four-dimensional computed tomography in haemorrhagic shock.

The 4D-VR images demonstrated the blood flow and organ perfusion. The contrast in the aorta was washed out rapidly at 0% occlusion but remained at the end of the scan at 100% occlusion. The inferior vena cava (IVC) enhancement was delayed in the high degree occlusion (80 and 100%), but retrograde flow was not observed. Enhancement of the haptic and portal veins was not clearly demonstrated in the high degree of occlusion (Fig. 1, Supplement).

**Figure 2:**
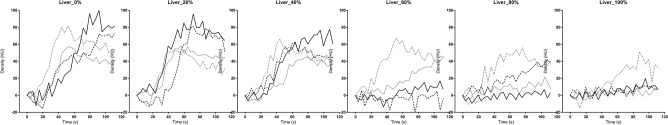
Time-density curve of the liver parenchyma under a regulated occlusion volume in partial resuscitative endovascular balloon occlusion of the aorta. The peak density of the time-density curve gradually decreased, and the time to peak was increasingly delayed as balloon volume increased.

**Figure 3:**
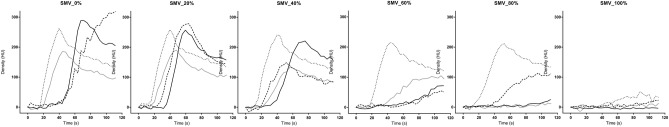
Time-density curve of the superior mesenteric vein under a regulated occlusion volume in partial resuscitative endovascular balloon occlusion of the aorta. The peak density of the time-density curve gradually decreased, and the time to peak was increasingly delayed as balloon volume increased.

**Figure 4:**
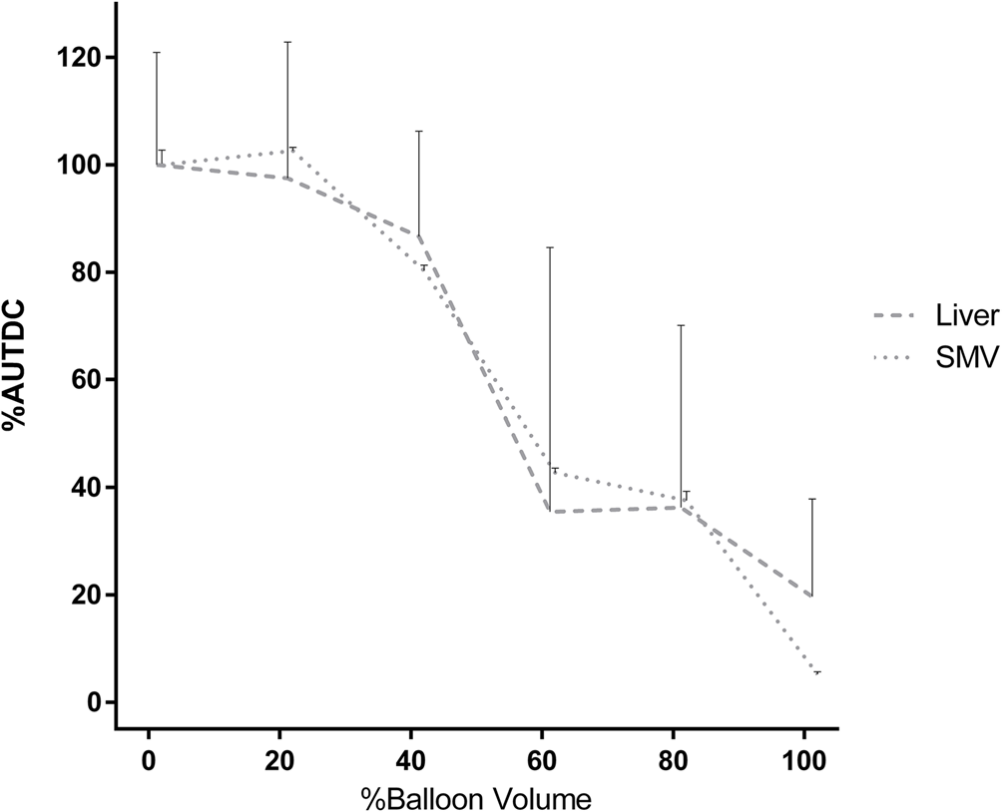
The change rate of the area under the time-density curve (AUTDC) during regulated occlusion volume in partial resuscitative endovascular balloon occlusion of the aorta. The AUTDC of the liver and the superior mesenteric vein (SMV) decreased linearly from 0 to 100% (liver, Y = −0.91*X + 108, R^2^ = 0.90, P = 0.0043; SMV, Y = −1.0*X + 112, R^2^ = 0.94, P = 0.0016).

The TDC of the liver parenchyma and SMV showed similar patterns. The peak density decreased and the time-to-peak was delayed as the occlusion degree increased from 0 to 80%. The TDC did not show a clear peak density at 100% occlusion (Figs. 2, 3). The change rate of AUTDC of the liver and SMV decreased linearly as the percent balloon volume increased (PV, Y = −1.071*X + 106.8, r^2^ = 0.972, P = 0.0003; liver, Y = −1.050*X + 101.8, r^2^ = 0.933, P = 0.0017; SMV, Y = −0.985*X + 100.3, r^2^ = 0.952, P = 0.0009) (Fig. 4).

## Discussion

This study continues and expands on a previous study that evaluated organ ischemia during P-REBOA in euvolemic status. 4D-CT has been used to assess cerebral, myocardial, or visceral organ perfusion^16–19^. First, we visually evaluated the 4D-VR to interpret changes in blood flow. We then analyzed the TDC to understand changes in organ perfusion according to the degree of occlusion.

As observed in the non-haemorrhagic state, the aortic flow was interrupted and the enhancement of the aorta remained longer with a high degree of occlusion. The 4D-VR images were visualized and interrupted by washout of the contrast in the aorta, in both the non-haemorrhage and haemorrhagic shock models. In comparison with the previous study, there was a significant absence of retrograde flow of the IVC in high-degree occlusion. Although high-degree occlusion induced IVC congestion and retrograde IVC flow in the euvolemic status, REBOA in haemorrhagic shock may be used without IVC congestion to control aortic flow.

In the TDC analyses for the liver and SMV, a reasonable and convincing change was observed in the transition of the curve, which decreased as the occlusion degree became stronger. This trend was similar to that observed under the non-bleeding conditions. The TDC of the liver and SMV described the changes in liver and bowel perfusion. However, the variability of the individual curves was more significant than that in the haemorrhagic shock condition. This variability may be due to the inability to accurately define the maximum balloon volume because of changes in the aortic diameter in haemorrhagic shock and individual differences.

AUTDC decreased linearly with percent balloon volume. The trend demonstrated a linear relationship between percent balloon volume and organ perfusion regardless of intravascular volume, suggesting the prediction of organ ischemia based on balloon volume. In the experimental model, adequate flow control during prolonged P-REBOA optimized bleeding control and decreased ischemic injury^20,21^. P-REBOA can be adjusted based on distal pressure to some extent^10,22^. Distal pressure measurement, which can be measured with a conventional arterial line or a portable handheld device (the COMPASS device; Mirador Biomedical, Seattle, WA)^23^, is a clinically available parameter for the titration of P-REBOA. However, distal pressure does not necessarily reflect distal arterial flow or organ perfusion^24^. Balloon volume can reflect distal organ perfusion and is a clinically usable parameter, which are the strengths of the P-REBOA titration using percent balloon volume.

Although dynamic 4D-CT is a unique and reproducible technique for quantitatively evaluating organ ischemia without surgical invasion, this study had several limitations. For example, more significant individual differences were observed during haemorrhagic shock than during nonbleeding since the AUTDC is a surrogate parameter for organ perfusion. Also, the experimental animals included only young female for the feasibility and safety of the experiment. Quantitative evaluations such as time-to-peak, peak value, and AUTDC are just a tiny part of the capabilities of CT perfusion. Despite these limitations, this study presents a novel scope for predicting organ ischemia. Further investigation in humans will reveal the clinical safety and evaluation of organ perfusion during P-REBOA.

## Conclusions

Dynamic 4D-CT revealed less severe IVC congestion during P-REBOA in haemorrhagic shock than in euvolemia. Analyses of TDC of the liver and SMV revealed a linear change in organ perfusion according to the percent balloon volume, regardless of intravascular volume.

## Supplementary Information

Supplementary Video 1.

Supplementary Legends.

## Data Availability

The datasets used and/or analyzed during the current study are available from the corresponding author on reasonable request.
