# Supplementary material for: Organ perfusion during partial REBOA in haemorrhagic shock: dynamic 4D-CT analyses in swine
Source: Sci Rep. 2022 Nov 5;12:18745. doi: 10.1038/s41598-022-23524-y (PMC9637200; doi:10.1038/s41598-022-23524-y)
Supplement: Supplementary file 2 — Supplementary Legends. [file 41598_2022_23524_MOESM2_ESM.docx]

**Supplemental Video.** Four dimensional-volume rendering images of the dynamic computed tomography data. The degree of occlusion increased from 0% (upper left) to 100 % (lower right). The contrast in the aorta was washed out rapidly at 0% occlusion but remained at the end of the scan at 100% occlusion. IVC enhancement was delayed in the high degree occlusion (80 and 100%), but retrograde flow was not observed. Enhancement of the haptic and portal veins was not clearly demonstrated by the high degree of occlusion.
